# Cardioprotective Properties of Kaempferol: A Review

**DOI:** 10.3390/plants12112096

**Published:** 2023-05-24

**Authors:** Yusof Kamisah, Juriyati Jalil, Nurhanan Murni Yunos, Satirah Zainalabidin

**Affiliations:** 1 Department of Pharmacology, Faculty of Medicine, Universiti Kebangsaan Malaysia, Kuala Lumpur 56000, Malaysia; 2 Centre for Drug and Herbal Development, Faculty of Pharmacy, Universiti Kebangsaan Malaysia, Kuala Lumpur 50300, Malaysia; juriyatijalil@ukm.edu.my; 3 Natural Products Division, Forest Research Institute of Malaysia, Selangor 52109, Malaysia; hanan@frim.gov.my; 4 Program of Biomedical Science, Centre of Applied and Health Sciences, Faculty of Health Sciences, Universiti Kebangsaan Malaysia, Kuala Lumpur 50300, Malaysia; satirah@ukm.edu.my

**Keywords:** cardiac disease, flavonoid, cardiac function, antioxidant, anti-inflammatory, antifibrosis, antiapoptosis, calcium regulation

## Abstract

Cardiac diseases, such as myocardial infarction and heart failure, have become a major clinical problem globally. The accumulating data demonstrate that bioactive compounds with antioxidant and anti-inflammatory properties have favorable effects on clinical problems. Kaempferol is a flavonoid found in various plants; it has demonstrated cardioprotective properties in numerous cardiac injury models. This review aims to collate updated information regarding the effects of kaempferol on cardiac injury. Kaempferol improves cardiac function by alleviating myocardial apoptosis, fibrosis, oxidative stress, and inflammation while preserving mitochondrial function and calcium homeostasis. However, the mechanisms of action of its cardioprotective properties remain unclear; therefore, elucidating its action could provide insight into directions for future studies.

## 1. Introduction

Cardiovascular diseases are the principal cause of mortality globally, claiming approximately 18 million lives each year [1]. Cardiac disease refers to conditions affecting the heart, including coronary artery disease and arrhythmia. In coronary artery disease, the blood supply that carries oxygen and nutrients to the heart is interrupted, which could be due to atherosclerotic plaque buildup. This may precipitate an ischemic condition in the heart, leading to myocardial infarction. In animal studies, the condition can be mimicked by the models of ischemia/reperfusion (I/R) produced by the ligation of coronary artery [2] or aorta [3], while in in vitro studies, this may be achieved by employing anoxia/reperfusion (or anoxia/reoxygenation) [4] and hypoxia/reperfusion models [5]. Myocardial infarction may precipitate the development of cardiac remodeling, which later could progress to heart failure. Cardiac remodeling can also be triggered by the administration of angiotensin II (Ang II) [6] and cardiotoxic drugs, including doxorubicin [7], cisplatin [8], 5-fluorouracil [9], and clozapine [10], in animal studies. Phenylephrine [3] and isoprenaline [11], which cause myocardial overstimulation, are also used to develop myocardial infarction and heart failure depending on the dose and duration of exposure [12]. Another complication of myocardial infarction is cardiac arrhythmia. Diabetes may also cause detrimental changes to the heart, known as diabetic cardiomyopathy, evidenced by increased oxidative stress, inflammation, apoptosis, and fibrosis [13].

Plants have a crucial role in human life and well-being. Humans use plants for food, clothing, furniture, and many other things. Plants have been used for medicinal purposes since ancient times. They produce secondary metabolites, such as flavonoids and terpenoids, for their self-defense [14]. Flavonoids are found abundantly in vegetables and fruits. The compounds are responsible for the pigmentation of yellow and red, as well as other colors in plants. They are divided into seven subclasses: flavonols (e.g., kaempferol and quercetin), flavones (e.g., luteolin and apigenin), flavanols (e.g., catechin and epicatechin), isoflavones (e.g., daidzein and genistein), anthocyanidins (e.g., cyanidin and delphinidin), flavonones (e.g., hesperetin and hesperidin), and chalcones (e.g., butein and naringenin chalcone) [15,16,17]. 

Flavonoids have been isolated from plants for various therapeutic effects. A study demonstrated that rutin and quercetin reduced Ang-II-induced cardiomyocyte hypertrophy by modulating mitogen-activated protein kinase (MAPK) [18]. On the other hand, kaempferol [11] and luteolin [19] protected against diabetic cardiomyopathy by regulating Kelch-like ECH-associated protein (Keap) and nuclear factor kappa B (NF-κB) signaling pathways. Hesperidin also diminishes injury following myocardial infarction in mice by modulating inflammatory response [20]. Therefore, plants have become a promising source of new drugs over the last four decades [21].

**Figure 1:**
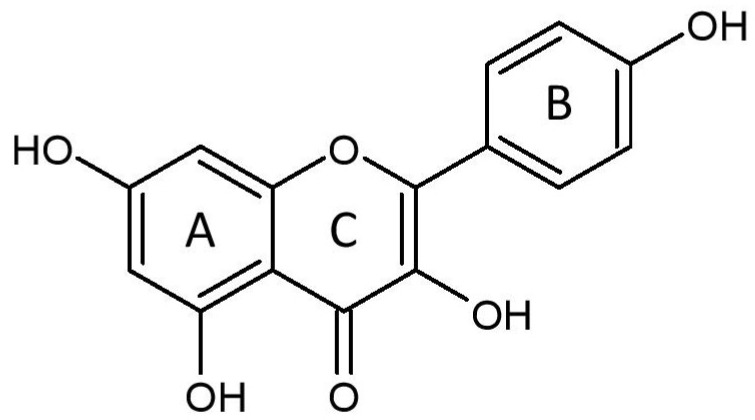
The molecular structure of kaempferol, consisting of benzene rings A and B, as well as a heterocyclic ring C.

Kaempferol (3,4′,5,7-tetrahydroxyflavone) (Figure 1), a yellow crystalline compound, is a flavonol which is rich in various plants such as tea, broccoli, tomatoes [22], and beans (e.g., bitter bean) [23]. Many studies have demonstrated the pharmacological activities of this compound in various pathological conditions, such as cardiovascular disease [6,13] and cancer [24]. It protects against cardiac disease via antiapoptotic, antioxidative, anti-inflammatory, calcium regulatory, and antifibrotic mechanisms, as well as maintaining mitochondrial function, resulting in the amelioration of cardiac structure and function [25,26,27]. However, the specific protective mechanisms of kaempferol remain unclear. The current review aims to provide an up-to-date overview of the role of kaempferol in cardiac disease. The information provided could increase the understanding of the cardioprotective effects of kaempferol and could aid in the design of future studies.

## 2. Bibliographic Search

A search of the literature was systematically performed using the PubMed, Scopus, and Web of Science databases. The keywords used for the search were “kaempferol” AND “cardiovascular”, “kaempferol” AND “cardiac”, “kaempferol” AND “heart”, “kaempferol” AND “myocardi*”, and “kaempferol” AND “cardiomyo*”. The search retrieved 41 articles that were published from 2008 to March 2023.

## 3. Effects on Cardiac Injury and Structure

Numerous models have been employed to determine the potential effects of kaempferol on cardiac injury. Various agents have been used in experimental animals and cardiomyocytes in vitro to induce cardiac injury, namely Ang II [6], isoprenaline [25], doxorubicin [7], cisplatin [8], 5-fluorouracil [9], phenylephrine [3], and clozapine [10], in addition to I/R [28] and aortic banding [3]. The inducers promote cardiac injury by elevating oxidative stress and inflammation, the culprits in cardiac injury. A burst of reactive oxygen species (ROS) production occurs upon the reintroduction of oxygen (reperfusion). Kaempferol reduces cardiac injury in animal models, as observed by the reduction in the release of cardiac injury markers, including creatine kinase, creatine kinase MB, troponin, and lactate dehydrogenase (Table 1). It also curbs myocardial infarct size in rat hearts that undergo I/R [27] and in rats exposed to isoprenaline [25]. Meanwhile, in rats receiving 5-fluorouracil, kaempferol post-treatment reduced myocardial inflammatory changes, namely hyaline formation, necrosis, and hyperemia [9]. The observed protective effects of kaempferol come from its antioxidant and anti-inflammatory properties, for which the details are discussed later. The antioxidant property of kaempferol is attributable to the presence of hydroxyl group on the B-ring (Figure 1) [29].

Kaempferol decreases levels of atrial natriuretic peptide (ANP) and B-type natriuretic peptide (BNP) [3,8,11]. Both peptides are released in response to volumetric stretch of the atrial and ventricular walls [34]. Structurally, it also diminishes myocardial fiber derangement induced by cisplatin [8]. It reduces the detrimental effects of heart inducers by reducing interventricular septal thickness at diastole (IVSD), left ventricular internal diameter (IVIDd), and posterior wall (LVPWd) in diastole and systole [3,30], indicating the ability of kaempferol to decrease left ventricular wall thickening, a common phenomenon in cardiac remodeling. A change in left ventricular geometry triggers its remodeling [35]. The findings propose that kaempferol possesses antihypertrophic activity in the myocardium, confirmed by a reduction in cardiomyocyte size and heart weight-to-body weight ratio [3,6,8,30]. The protective effects are primarily via its blockade of ROS synthesis [3], which prevents subsequent events. Many events are involved in the development of cardiac hypertrophy and remodeling including fibrosis, apoptosis, and altered mitochondrial function, and the effects of kaempferol on the events will be discussed later.

Excessive activity of renin-angiotensin system could predispose to the development of left ventricular hypertrophy [36]. However, studies investigating the effects of kaempferol on the aspect are still lacking. Ang II, a proinflammatory peptide, is a principal substance in the renin–angiotensin system that plays a principal role in the pathogenesis of hypertrophy [37]. Even though many studies have demonstrated the positive effects of kaempferol on Ang-II-induced hypertrophy, the effects of the bioactive compound on the expression of Ang II; the angiotensin-converting enzyme?the Ang II synthesis enzyme; and its receptor, Ang II type 1 receptor, have not been explored. Understanding the mechanisms could shed more light on its potential pharmacological activities.

The beneficial effects of the compound (10 mg/kg on alternate days for 8 weeks) were also observed in diabetic mice with cardiac injury. It decreased disorganization of myofiber and derangement of cellular structures in the diabetic heart [13]. It is unknown whether its protective effects were via reduction in glucose level since it was not measured in the study. However, kaempferol at higher doses (50−200 mg/kg) was reported to reduce the plasma glucose level in streptozotocin-induced diabetic rats after 15 days [38]. Hyperglycemia augments the production of ROS, leading to increased oxidative stress and inflammation, contributing to diabetic complications [39]. Therefore, it is possible that kaempferol protects the diabetic heart by bringing down the glucose level and thereafter preventing hyperglycemia-induced increases in oxidative stress and inflammation.

Taken together, these findings are suggestive of the ameliorative effects of the compound on heart structure, possibly via its antioxidant and anti-inflammatory properties. Via its anticardiac remodeling property, kaempferol could lessen exacerbating economic strain arises from comorbidities experienced by the patients. However, the impact of kaempferol on cardiac structure has not been extensively investigated. To the best of our knowledge, no clinical trial has been conducted to evaluate the effects of kaempferol on cardiovascular disease.

## 4. Effects on Cardiac Function

Kaempferol exhibits positive effects on cardiac function in various cardiac injury models. It improves left ventricular fractional shortening (LVFS) and ejection fraction (LVEF) (Table 2) [3,6,8,33]. Both parameters are used to detect left ventricular systolic function and are reduced in hearts with left ventricular failure [40,41]. Therefore, these parameters are used in the diagnosis of heart failure [42]. Other than the parameters, improvement of the systolic function by kaempferol (15 mM and 20 mg/kg/day) pretreatment are also evidenced by an increase in the maximal rate of rise (+dp/dt_max_) and fall (−dp/dt_max_) of left ventricular pressure in I/R- and isoprenaline-induced myocardial injury in rats [27,28,31,43], as well as left ventricular systolic pressure and developed pressure [2,27,32] in models of acute myocardial infarction and I/R injury. However, similar positive findings were not observed in left anterior descending coronary artery (LADCA)-ligation-induced heart failure in mice receiving 12 mg/kg/day for 3 days [44], possibly due to the shorter duration of kaempferol treatment compared with other studies. 

The beneficial effects of the flavonoid were also observed in diastolic function. It reduces left ventricular end-diastolic pressure (LVEDP; see Table 2) [2,30,31,32,43]. LVEDP measures left ventricular preload and diastolic compliance [45], indicating that kaempferol reduces preload, which is useful in angina. The reduction in the ratio of transmitral flow velocity/mitral annular velocity and the increase in left ventricular volume of diastole by kaempferol treatment indicate an improvement in myocardial diastolic function [3,6]. The betterment in diastolic function by kaempferol eventually indirectly improves the systolic function.

Kaempferol possibly protects by preventing the loss of contractile function due to reducing the number of viable cardiomyocytes following an insult to myocardium, thereby hindering the development of cardiac remodeling. Consequently, myocardial inotropic and lusitropic properties are preserved by kaempferol. The restoration of both properties is crucial in maintaining a normal cardiac performance. Cardiac function is partly determined by myocardial cellular and molecular structures, including calcium regulators and mitochondrial function. Ca^2+^ is required for myocardial contraction, while mitochondria functions to supply energy for myocardial activities [46]. However, increased production of ROS can perturb the function of the components. Therefore, the capability of kaempferol in scavenging ROS plays a prominent part in its protective role.

Excessive activation of sympathetic nervous and renin–angiotensin systems may augment the risk of cardiac dysfunction [36]. Raised myocardial epinephrine concentration is associated with increased resting heart rate in rats with failing hearts [47]. However, it is unknown whether kaempferol has any effects on epinephrine level or β1-adrenoceptor in the heart that may somewhat contribute to its cardioprotective effects.

Collectively, kaempferol can improve myocardial left ventricular systolic and diastolic function and prevent the development of arrhythmia. However, studies to date have only examined the effect of kaempferol on left ventricular function; no study has investigated its effect on right ventricular function. Future studies should focus on this aspect.

## 5. Effects on Myocardial Calcium Regulation and Rhythm

Cardiac calcium regulation is closely linked to cardiac contraction. The sarcoplasmic reticulum is the main source of calcium for myocardial contraction [48]. Calcium regulatory proteins such as the sodium–calcium exchanger (NCX), Ca^2+^-ATPase, and sodium–potassium ATPase (Na^+^/K^+^-ATPase) govern the uptake and release of calcium ions across cell membrane [49]. A reduction in intracellular Na^+^ triggers NCX activity to bring more Na^+^ inside the cells in exchange for Ca^2+^ [49], while Ca^2+^-ATPase regulates the uptake of Ca^2+^ into cells and the sarcoplasmic reticulum (known as sarcoplasmic endoplasmic reticulum Ca^2+^-ATPase (SERCA)) [48]. Kaempferol (100 mg/kg) post-treatment for 45 days reversed the reduction in myocardial membrane-bound Na^+^/K^+^-ATPase, Ca^2+^-ATPase, and total ATPase activity in diabetic rats (Table 3) [26]. The findings suggest that kaempferol maintains cardiac calcium homeostasis and safeguards the integrity of the membrane under pathological conditions. The effects of kaempferol on other calcium regulators, such as NCX and ryanodine receptor type 2 (RyR2) for Ca^2+^ release and phospholamban, which is involved in Ca^2+^ uptake, are yet to be studied. Understanding these aspects could shed light on the mechanisms of action of kaempferol.

**Figure 2:**
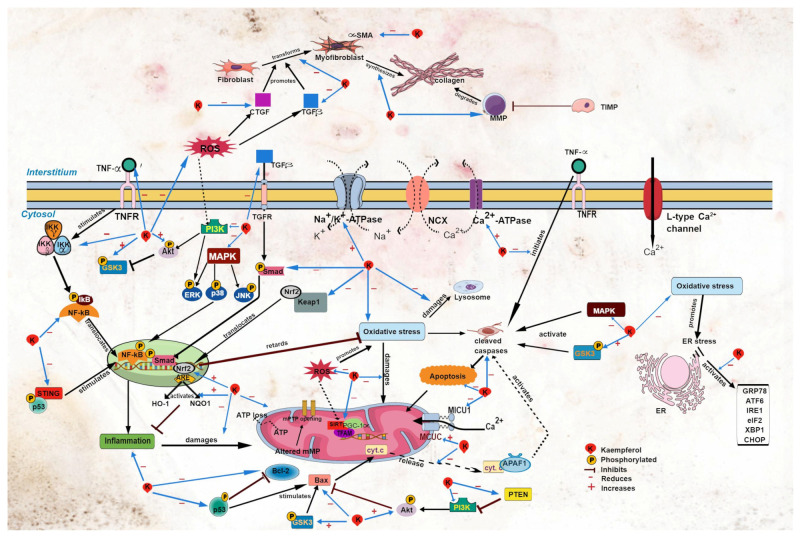
Sites of action of kaempferol in myocardial injury. Akt, protein kinase B; APAF1, apoptotic protease activating factor 1; ARE, antioxidant response element; ATF6, activating transcription factor 6; CHOP, C/EBP homologous protein; CTGF, connective tissue growth factor; cyt. c, cytochrome c; eIF2, eukaryotic initiation factor 2α; ER, endoplasmic reticulum; ERK, extracellular signal-regulated kinase; GRP78, glucose regulatory protein 78; GSK3, glycogen synthase kinase-3; HO-1, heme oxygenase-1; IKB, inhibitor of κB kinase; NF-κB, nuclear factor kappa B; IKKα, inhibitor of NF-κB kinase α; IKKβ, inhibitor of NF-κB kinase β; IKKγ, inhibitor of NF-κB kinase γ; IRE1, inositol-requiring transmembrane kinase endoribonuclease-1α; JNK, c-Jun N-terminal kinase; Keap1, Kelch-like ECH-associated protein 1; MAPK, mitogen-activated protein kinase; MCUC, mitochondrial Ca^2+^ uniporter complex; MICU1, mitochondrial Ca^2+^ uptake 1; MMP, matrix metalloproteinase; mPTP, mitochondrial permeability transition pore; NQO1, NAD(P)H dehydrogenase (quinone 1); Nrf2, nuclear factor erythroid 2 p45-related factor 2; p38, p38 MAPK; p53, p53 tumor suppressor gene; PGC-1α, peroxisome proliferator-activated receptor-γ coactivator 1α; PI3K, phosphoinositide 3-kinase; PTEN, phosphatase and tensin homolog; ROS, reactive oxygen species; SIRT1, silent information regulator type 1; α-SMA, α-smooth muscle actin; Smad, small mothers against decapentaplegic; STING, stimulator of interferon genes; TFAM, mitochondrial transcription factor A; TGFβ, transforming growth factor β; TIMP; TNFα, tumor necrosis factor α; TNFR, tumor necrosis factor receptor; XBP1, X-box binding protein 1.

Altered Ca^2+^ homeostasis can provoke cardiac arrhythmias, such as atrial fibrillation. In patients with metabolic syndrome, cardiac mitochondrial Ca^2+^ uniporter complex (MCUC) is downregulated, resulting in impaired mitochondrial Ca^2+^ uptake and leading to atrial fibrillation [50]. Kaempferol treatment protects against cardiac arrhythmia; it protects against the development of sinus node dysfunction [51] and ventricular arrhythmia in mice following LADCA ligation (Table 3) [50]. Kaempferol augments mitochondrial Ca^2+^ uptake [44,53,54] by restoring MCUC activity, and it abolishes atrial fibrillation in obese diabetic mice [50]. In addition, kaempferol may decrease the expression of mitochondrial Ca^2+^ uptake 1 (MICU1), a MCUC gatekeeper [55], leading to the augmented uptake of the ion by mitochondria (Figure 2). This postulation should be confirmed in future studies. Ca^2+^ is needed for mitochondrial ATP production to provide energy for contractile proteins [46,56]. Therefore, the increased mitochondrial uptake of Ca^2+^ induced by kaempferol suggests the amelioration of mitochondrial energy metabolism and cardiomyocyte function.

In heart failure, intracellular diastolic Ca^2+^ levels are increased [44], preventing ventricular relaxation and blood refill [57]. In various models of cardiac hypertrophy, kaempferol (10 μM) diminished diastolic Ca^2+^ waves and sparks [41,44,54]. The findings suggest that kaempferol improves myocardial diastolic function, as demonstrated in other studies [3,6]. However, a similar protective effect of kaempferol (1 μM) was not observed in isolated hearts obtained from mice with thoracic aortic banding following isoprenaline administration [52]. In the study, kaempferol increased the rate of Ca^2+^ accumulation in the mitochondria, leading to an increased production of spontaneous Ca^2+^ waves, likely due to increased release of the ion from the sarcoplasmic reticulum. Similar findings were observed in primary cardiomyocytes isolated from mice, using 10 μM kaempferol [52]. The discrepancy is unexplainable, and it should be investigated further.

## 6. Effects on Cardiac Oxidative Stress and Inflammation

Oxidative stress and inflammation play a prominent role in various diseases, including cardiac disease [58]. ROS are implicated in oxidative stress [59] and inflammation [60,61]. Kaempferol protects the heart by reducing lipid peroxidation products (e.g., thiobarbituric acid reactive substance, malondialdehyde, conjugated diene, and lipid hydroperoxide); increasing antioxidant enzymes, namely superoxide dismutase, catalase, and glutathione peroxidase, in diabetic rats [5,31,38]; and decreasing pro-inflammatory cytokines (e.g., tumor necrosis factor α (TNFα) and interleukin 6) (Table 4) [6,8]. These findings confirm its antioxidant and anti-inflammatory properties.

Nuclear factor erythroid 2 p45-related factor 2 (Nrf-2) is a transcription factor that modulates oxidative stress [64]. Upon activation, Nrf2 dissociates from its complex with Keap1 (Figure 2). Activated Nrf2 governs the antioxidant response by upregulating the expression of its downstream effectors, namely heme oxygenase-1 (HO-1) and NAD(P)H dehydrogenase (quinone 1) (NQO1) [64,65]. Upregulation of these antioxidant factors (Nrf2, HO-1, and NQO-1) by kaempferol was observed in various models of heart disease, including diabetic cardiomyopathy [11,13], high-glucose-induced cardiomyocyte injury [13], and Ang-II-induced cardiomyocyte hypertrophy [6]. Kaempferol also downregulates the *Keap1* gene [11], which is suggestive of its ability to increase the activation of the antioxidant signaling pathway.

NF-κB is a principal inflammatory regulator that regulates chemokines and pro-inflammatory cytokines. To function, it needs to be activated prior to its translocation to the nucleus. This activation is strictly governed by the inhibitor of the κB kinase (IκBα and IκBβ) and inhibitor of the NF-κB kinase (IKKα and IKKβ) [66]. Kaempferol downregulates the TNFα and NF-κB expression and upregulates its inhibitory molecules (IκBα and IKKβ) [2,6,8,11,13,28,62] in models of cardiac disease (Table 4 and Figure 2). TNFα is a stimulator of NF-κB. The findings of these studies suggest that kaempferol diminishes pro-inflammatory stimuli, leading to the upregulation of inhibitory molecules for NF-κB, thereby preventing translocation of NF-κB into the nucleus.

The MAPK and phosphoinositide 3-kinase/protein kinase B/glycogen synthase kinase-3β (PI3K/Akt/GSK3β) signaling pathways are involved in the modulation of oxidative stress and inflammation. MAPK has three subfamilies: extracellular signal-regulated kinase (ERK), c-Jun N-terminal kinase (JNK), and p38 MAPK (p38) [18]. In experimental cardiac disease models, kaempferol inhibits the activation of the MAPK subfamilies [3,6,7,11,28,31], preventing their nuclear translocation, thereby inhibiting the activation of diverse transcription elements. Furthermore, kaempferol inhibits the activation of the PI3K/Akt/GSK3β signaling pathway [11,27,62].

Ca^2+^/calmodulin-dependent protein kinase II (CaMKII) is a calcium-handling protein. It enhances calcium reuptake into the sarcoplasmic reticulum by SERCA [67]. However, under pathological conditions, CaMKII can become oxidized. In mice with Ang-II-induced sinus node dysfunction, oxidized CaMKII protein expression was significantly elevated; however, oxidation of the protein was inhibited by simultaneous treatment with kaempferol [51], indicating that the flavonoid conserves the activity of the handling protein via its antioxidant mechanism.

Despite the various protective effects of kaempferol against oxidative stress and inflammation, Hamilton et al. [52] reported that kaempferol (10 μmol/L) increased ROS production in the mitochondria of cardiomyocytes isolated from mice with cardiac hypertrophy that underwent arrhythmia induction using isoprenaline. The concentration of kaempferol used was in line with other studies [4,5,10]. The discrepancy is unexplainable until further investigations are performed.

Taken together, kaempferol exerts antioxidant and anti-inflammatory properties in numerous models of cardiac pathology by modulating Nrf2, NF-κB, MAPK, and PI3K/Akt/GSK3β signaling pathways. However, the detailed mechanisms of its antioxidant and anti-inflammatory properties in pathological cardiac events are unclear. The effects of kaempferol on the calcineurin/nuclear factor of activated T cells (NFAT) inflammatory signaling pathway should also be investigated.

## 7. Effects on Cardiac Mitochondrial Function Other Organelle Damage

Mitochondria, the cellular powerhouse, are vital in all cells, including cardiomyocytes, for generating energy for cellular functions [68]. The mitochondrial membrane potential plays a crucial role in storing energy during oxidative phosphorylation. Disturbed potential production, such as in the presence of increased ROS production, may cause mitochondrial dysfunction. This results in the opening of the mitochondrial permeability transition pore (mPTP) and ATP loss [69,70].

Studies have demonstrated that kaempferol reduces mPTP opening [4,52] and mitochondrial membrane potential collapse in clozapine- [10] and doxorubicin-induced [7] cardiac injury and in myocardial anoxia/reperfusion injury (Table 5) [4]. Another indicator for mitochondrial damage is the cytosolic release of cytochrome c, an enzyme found in mitochondrial inner membranes [27]. Kaempferol reduces myocardial cytochrome c release in I/R-induced cardiac injury in rats [27] and in cardiomyocytes exposed to doxorubicin [7] and anoxia/reperfusion (Figure 2) [4], suggestive that the compound preserves the integrity of the mitochondrial membranes. The protective effects of kaempferol are likely due to its ROS-scavenging activity; elevated ROS production and oxidative stress were observed in the studies. Suppression of the oxidative events by kaempferol decreases the mitochondrial membrane damage observed by a reduction in membrane permeability. Accordingly, kaempferol enhances the function of mitochondria by promoting their uptake of Ca^2+^ [53,54] for ATP synthesis, which is needed to supply the contractile proteins [56]. This results in a rise of ATP production and amelioration of mitochondrial metabolic capacity. These events preserve potential generation and improve mitochondrial function for energy storage, leading to improvement in the cardiac function by kaempferol.

Human silent information regulator type 1 (SIRT1) is a nuclear protein which has a role in mitochondrial biogenesis and turnover. Together with its substrate, peroxisome-proliferator-activated receptor gamma coactivator-1α (PGC-1α), SIRT regulates mitochondrial energy metabolism. It also governs mitochondrial longevity by promoting the mitophagy of damaged mitochondria [71]. Kaempferol raises the expression of SIRT1 in cardiomyocytes exposed to anoxia/reperfusion [4]. The addition of sirtinol, a SIRT1 inhibitor, impedes the ROS-inhibiting effect, mPTP opening suppression, and mitochondrial membrane potential restoration by kaempferol [4]. The findings confirm the involvement of SIRT1 in the cardioprotection conferred by kaempferol, which then boosts the metabolic function and longevity of mitochondria.

Exposure to stress such as I/R causes disproportioned proteostasis at the endoplasmic reticulum (ER) that results in an accumulation of nonfunctional misfolded proteins. This will trigger ER stress via the unfolded protein response (UPR) signaling pathway to promote cellular repair. Activating transcription factor 6α (ATF6α), protein kinase RNA-like ER kinase (PERK), and inositol-requiring transmembrane kinase endoribonuclease-1α (IRE1α) are ER stress sensors which interact with glucose-regulated protein 78 (GRP78) [72]. The accumulation of misfolded proteins causes dissociation of GRP78 from the stress sensors [73]. Kaempferol protects against the activation of ATF6α, IRE1α, GRP78, X-box binding protein 1 (XBP-1), and eukaryotic initiation factor 2α (eIF2α) in cardiomyocytes exposed to I/R (Table 5) [32]. XBP-1 is spliced by IRE1α before being translocated to the nucleus, while eIF2α is activated by PERK [73]. Another downstream molecule that is activated in ER stress is C/EBP homologous protein (CHOP), the activation of which is also reduced by kaempferol [32]. Activation of the ER stress proteins will ultimately inhibit the initiation of global protein translation [73]. The findings propose that kaempferol provides protection via its antioxidant and anti-inflammatory activities by diminishing potential stress to ER, thereby preventing the activation of the UPR signaling pathway. The activation of the stress sensors that trigger GRP78 dissociation is then halted by kaempferol. Finally, the synthesis of nonfunctional proteins is decreased. Thus, it can be concluded that kaempferol conserves functional protein synthesis.

Another organelle that is important in cellular function is lysosome. It has a role in cellular protein trafficking via non-classical protein secretion, which is independent of the ER and Golgi apparatus [73], in addition to its degradative function [74]. Kaempferol decreases lysosomal membrane instability [10], in line with the decreased oxidative stress, inflammation, and apoptosis. Indirectly, kaempferol increases lysosomal survivability and, hence, restores the functions of the organelle.

Therefore, kaempferol can protect organelles against oxidative-induced damage. It preserves mitochondrial, lysosomal, and ER membrane integrity, thereby maintaining cellular functions. Other aspects, such as mitochondrial transcription factor A (TFAM), which has a role in mitochondrial replication and energy generation [75]; and fission 1 (FIS1) and optic atrophy 1 (OPA1), which are involved in mitochondrial fission and fusion [76], respectively, could be examined to better comprehend the effects of kaempferol on mitochondrial function.

## 8. Effects on Cardiac Apoptosis

Accumulating evidence demonstrates the anti-apoptotic properties of kaempferol in various models of cardiac disease. Apoptosis, a programmed cell death that is elevated in experimental cardiac diseases, has a crucial role in mitochondrial function and is associated with excessive ROS production [4,5,33]. Kaempferol diminishes the expression of major protease effectors caspase 1 and caspase 3, thereby reducing the number of apoptotic cells (Table 6) [2,4,5,7,11,27,28,31,32,51]. The protective effects of the flavonoid are extended to a reduction in apoptotic DNA fragmentation, evidenced by decreased poly(ADP-ribose)polymerase (PAPR) cleavage and terminal deoxynucleotidyl transferase dUTP nick-end labeling (TUNEL)-positive cells [2,7,8,11,13,51]. Kaempferol exerts its protective effects by inhibiting the release of cytochrome c into the cytosol [4,7,21], thereby preventing the formation of cytochrome c-apoptotic protease activating factor 1 complex. The complex activates caspase activity (Figure 2) [77]. Caspase activation can be initiated by TNFα binding to its receptors [78], suggesting that the suppressive effects of kaempferol on TNFα expression contribute to caspase inactivation.

Transcription of the *p53* tumor suppressor gene (*p53*) initiates apoptosis by suppressing B-cell lymphoma 2 (Bcl-2) and stimulating Bcl-2-associated X protein (Bax), which are both apoptosis-regulating proteins. Bcl-2 plays a role in inhibiting apoptosis, while Bax promotes apoptosis (pro-apoptosis) [78]. Kaempferol normalizes apoptosis in cardiac diseases by decreasing the expression of p53 and Bax, while increasing the expression of Bcl-2 (Table 6) [4,5,7,8,11,13,32,33,43], likely by blocking the activation of Akt/GSK-3β and p38/ERK signaling pathways [11]. This process is aided by the crosstalk between apoptosis, inflammation, and oxidative stress, whereby kaempferol suppresses the stimulator of interferon genes (*STING*)/NF-κB [8] and modulates the Notch1/phosphatase and tensin homolog/Akt (Notch1/PTEN/Akt) signaling pathways [5], leading to a decrease in apoptosis. MicroRNA21 (miR-21) plays a role in the cardioprotective effects of kaempferol. Inhibition of miR-21 abolishes the effects of the flavonoid on the Notch1/PTEN/Akt pathway [5].

Overall, kaempferol inhibits apoptosis via its antioxidant and anti-inflammatory properties, as both oxidative stress and inflammation can trigger apoptosis through crosstalk between various signaling pathways. Other apoptosis-related signaling pathways, including the survivor activating factor enhancement (SAFE) pathway [79] and TNF-related apoptosis-inducing ligand (TRAIL) pathway [78], should be explored to investigate the potential modulatory effects of kaempferol on these pathways.

## 9. Effects on Myocardial Fibrosis

Cardiac fibrosis is one of the earlier events that persists during cardiac remodeling. Fibrosis distorts the function of the heart, leading to the development of heart failure [80]. It occurs due to altered extracellular matrix regulation during inflammation, including increased degradation of the extracellular matrix by matrix metalloproteinases (MMPs), resulting in the accumulation in the interstitial space [81]. In fibrotic tissues, fibronectin and collagen types 1 and 3—the major components of the extracellular matrix?are upregulated [82]. Kaempferol attenuates the accumulation of MMPs in rats with isoprenaline-induced cardiac remodeling [25] and transforming growth factor β1 (TGFβ1)-induced fibrosis [30], eliciting a reduction in fibrosis (Table 7 and Figure 2) [2,6,13]. Furthermore, it abates the expression of collagen types 1, 3, and 4 in cardiomyocytes and cardiac fibroblasts exposed to high glucose [13], TGFβ1 [30], or Ang II [6]. These findings were confirmed in in vivo models of diabetic cardiomyopathy [13], Ang-II-induced cardiac dysfunction [30], and aortic-banding-induced cardiac remodeling [3]. Left ventricular collagen volume is significantly diminished following kaempferol treatment [2,3,30]. These observations are suggestive of its antifibrotic properties.

Myocardial fibrogenesis is regulated by growth factors such as TGFβ1 and connective tissue growth factor (CTGF), as well as the small mothers against decapentaplegic (Smad) signaling pathway [83]. Cardiac insult triggers the binding of active TGFβ1 to its receptor (TGFR), stimulating collagen synthesis via the Smad signaling pathway [84]. Furthermore, TGFβ1 enhances the conversion of fibroblasts into myofibroblast, marked by the presence of α-smooth actin (α-SMA) [46], the expression of which is downregulated by kaempferol (Table 7) [30]. The expression of the growth factors and phosphorylated Smad was significantly downregulated by kaempferol in various models of cardiac dysfunction [3,6,13,30]. The observed effects of kaempferol indicate that the flavonoid curtails the signaling pathway of collagen synthesis.

MMP degradation is regulated by the tissue inhibitor of metalloproteinase (TIMP); however, studies investigating the effects of kaempferol on TIMP are lacking. Other signaling mechanisms, such as the wingless-related integration site (Wnt)/β-catenin [81,85] and Hippo-Yes-associated protein/transcriptional coactivator with PDZ-binding motif (Hippo-YAP/TAZ) [86], are also involved in the pathogenesis of myocardial fibrosis. Kaempferol may modulate these signaling pathways.

## 10. Conclusions and Directions for Future Studies

Kaempferol is a natural antioxidant and anti-inflammatory which can be abundantly found in many plants. Increasing data from animal and in vitro studies have demonstrated the cardioprotective role of kaempferol. It demonstrates beneficial effects on cardiac structure and function. Most of the studies exhibit its prominent anticardiac remodeling. It protects against cardiac hypertrophy and remodeling in various experimental cardiac diseases via its regulation of myocardial calcium level, apoptosis, mitochondrial function, oxidative stress, inflammation, and extracellular matrix assembly. It also displays antiarrhythmic activity, which might be beneficial in patients suffering from myocardial infarction. The findings support its potential as a promising candidate for managing cardiac diseases. However, its effects on congenital cardiac disease and acquired heart diseases in pregnancy are yet to be examined.

Another aspect that could be explored is the impact of kaempferol on autophagy, a mechanism that is also involved in the pathogenesis of diverse cardiac diseases. Clinical studies should be conducted to confirm the protective effects of kaempferol that were observed in laboratories.

## Figures and Tables

**Table 1:** Effects of kaempferol on cardiac structure and injury biomarkers.

Animals/Cells	Model	Dose and Duration of Kaempferol	Findings	Reference
Mice	Aorta-banding-induced cardiac remodeling	100 mg/kg/d (i.g.) for 6 weeks	↓ ANP gene, ↓ BNP gene ↓ cardiomyocyte size ↓ HW/BW, ↓ LVIDd ↓ LVIDs, ↓ LVPWd ↓ LVPWs	[3]
H9c2 cardiomyocytes	Phenylephrine-induced cell hypertrophy	25 µM for 2 h (pretreatment)	↓ cardiomyocyte size	[3]
Rat cardiomyocytes	Anoxia/reperfusion	10, 20 or 40 μM for 24 h (pretreatment)	↓ LDH ↑ cell viability	[4]
H9c2 cardiomyocytes	H/R-induced injury	5, 10, 20, or 30 μM for 2 h (pretreatment)	↑ cell viability ↓ LDH	[5]
Mice	Ang-II-induced cardiac remodeling	10 mg/kg (p.o) on alternate days for 4 weeks	↓ cardiomyocyte size ↓ NT-proBNP, ↓ titin	[6]
Rats	Doxorubicin-induced cardiac injury	1, 2, 5, 10, and 20 mg/kg/d on alternates for 3 injections (i.p.) (pretreatment)	↑ BW, ↑ HW ↔ HW/BW ↓ serum LDH	[7]
H9c2 cardiomyocytes	Doxorubicin-induced injury	5, 10, 20, and 50 μM for 4 h (pretreatment)	↑ cell viability ↓ LDH	[7]
Mice	Cisplatin-induced cardiotoxicity	10 mg/kg/d (p.o.) for 2 weeks (pretreatment)	↓ BNP gene ↓ ANP gene ↓ serum CK-MB ↓ HW/BW	[8]
Rats	5-Fluorouracil-induced cardiotoxicity	1 mg/kg/d (i.p.) for 14 days (post-treatment)	↓ serum CK-MB ↓ serum LDH ↓ cardiac necrosis ↓ cardiac hyaline ↓ cardiac hyperemia	[9]
Rat cardiomyocytes	Clozapine-induced cardiac injury	10, 20, and 50 μM for 4 h	↑ cell viability	[10]
Diabetic rats	ISO-induced heart failure	10 and 20 mg/kg/d (p.o.) for 42 days (pretreatment)	↓ HW/BW, ↓ serum LDH ↓ serum troponin I ↓ serum CK-MB, ↓ serum BNP	[11]
Mice	STZ-induced diabetes	10 mg/kg (p.o.) on alternate days for 8 weeks (post-treatment)	↓ serum CK ↓ serum CK-MB ↓ serum LDH	[13]
Rats	ISO-induced cardiac injury	3 and 10 mg/kg/d for 7 days (pretreatment)	↓ infarct area ↓ serum LDH ↓ serum troponin I ↓ serum CK-MB	[25]
Rats	I/R-induced cardiac injury (Langendorff isolated heart)	15 mmol/L for 15 min (pretreatment)	↓ CK, ↓ LDH ↓ infarct size	[27]
Diabetic rats	I/R-induced cardiac injury	20 mg/kg/d (i.p.) for 28 days (pretreatment)	↓ serum LDH ↑ serum CK-MB	[28]
Mice	Ang-II-induced cardiac dysfunction	10 mg/kg (i.p.) for 2 weeks	↓ IVSD, ↓ HW/BW ↓ HW/TL ↓ cardiomyocyte size ↓ ANP gene, ↓ BNP gene ↓ vimentin protein	[30]
Rats	I/R-induced myocardial injury	20 mg/kg/d (i.p.) for 15 days (pretreatment)	↓ serum LDH ↑ serum CK-MB	[31]
H9c2 cardiomyocytes	I/R-induced injury	10 μM (pretreatment)	↑ cell viability	[32]
H9c2 cardiomyocytes	I/R-induced injury	5 μM for 24 h (pretreatment)	↑ cell viability	[33]

Ang II, angiotensin II; ANP, atrial natriuretic peptide; BNP, B-type natriuretic peptide; BW, body weight; CK-MB, creatine kinase MB; H/R, hypoxia/reperfusion; HW, heart weight; i.g., intragastric; i.p., intraperitoneal; I/R, ischemia/reperfusion; ISO, isoprenaline; IVSD, interventricular septal thickness at diastole; IVSTd, left ventricular internal diameter at diastole; IVSTs, left ventricular internal diameter at systole; LDH, lactate dehydrogenase; LVIDd, left ventricular internal diameter at diastole; LVIDs, left ventricular internal diameter at systole; LVPWd, left ventricular posterior wall dimension at diastole; LVPWs: left ventricular posterior wall at systole; NT-proBNP, N-terminal pro b-type natriuretic peptide; p.o., per oral; STZ, streptozotocin; TL, tibial length; ↑, increased; ↓, reduced; ↔, no change.

**Table 2:** Effects of kaempferol on cardiac function.

Animals	Model	Dose and Duration of Kaempferol	Findings	Reference
Rats	Acute myocardial infarction	10 mg/kg (i.g.) for 4 weeks	↓ LVSP, ↓ LVEDP ↑ +dp/dt_max_, ↓ −dp/dt_max_	[2]
Mice	Aorta-banding-induced cardiac remodeling	100 mg/kg/d (i.g.) for 6 weeks	↓ LVVd, ↓ LVVs, ↑ LVFS, ↑ LVEF	[3]
Mice	Ang-II-induced cardiac remodeling	10 mg/kg (p.o.) on alternate days for 4 weeks	↑ LVFS, ↑ LVEF, ↓ E/E′	[6]
Mice	Cisplatin-induced cardiotoxicity	10 mg/kg/d (p.o.) for 2 weeks (pretreatment)	↑ LVEF, ↑ LVFS	[8]
Rats	I/R-induced cardiac injury	15 mmol/L for 15 min (pretreatment)	↑ LVDP, ↑ +dp/dt, ↑ −dp/dt ↑ CF, ↑ HR	[27]
Diabetic rats	I/R-induced cardiac injury	20 mg/kg/d (i.p.) for 28 days (pretreatment)	↑ +dp/dt_max_, ↑ −dp/dt_max_ ↓ LVEDP	[28]
Mice	Ang-II-induced cardiac dysfunction	10 mg/kg (i.p.) for 2 and 4 weeks	↓ LVEDD, ↔ LVEF ↔ LVESD (2 weeks) ↓ LVESD (4 weeks)	[30]
Rats	I/R-induced myocardial injury	20 mg/kg/d (i.p.) for 15 days (pretreatment)	↓ LVEDP, ↑ +dp/dt_max_ ↑ −dp/dt_max_	[31]
Langendorff isolated heart	I/R injury	10 μM for 20 min pre-ischemia and 50 min post-ischemia	↑ LVDP, ↓ LVEDP	[32]
Mice	I/R-induced cardiac injury	10 mg/kg (pretreatment)	↑ LVEF, ↑ LVFS	[33]
Mice	LADCA-ligation-induced HF	12 mg/kg/d for 3 days	↔ LVSP, ↔ LVEDP ↔ +dp/dt_max_, ↔ −dp/dt_min_	[44]
Rats	ISO-induced cardiac injury	5, 10, and 20 mg/kg/d (i.p.) for 15 days (pretreatment)	20 mg/kg/d: ↑ +dp/dt_max_, ↑ −dp/dt_max_ ↓ LVEDP	[43]

Ang II, angiotensin II; AP, action potentials; CF, coronary flow; −dp/dt_max_, maximal rate of fall of left ventricular pressure; +dp/dt_max_, maximal rate of rise in left ventricular pressure; E/E′, ratio of early diastolic transmitral flow velocity and mitral annular velocity, transmitral flow velocity/mitral annular velocity; HF, heart failure; i.g., intragastric; i.p., intraperitoneal; I/R, ischemia/reperfusion; ISO, isoprenaline; LADCA, left anterior descending coronary artery; LVDP, left ventricular developed pressure; LVEDD, left ventricular end-diastolic dimension; LVESD, left ventricular end-systolic dimension; LVEDP, left ventricular end-diastolic pressure; LVEF, left ventricular ejection fraction; LVFS, left ventricular fractional shortening; LVSP, left ventricular systolic pressure; LVVd, left ventricular volume at diastole; LVVs, left ventricular volume at systole; p.o., per oral; PVC, premature ventricular contractions; VF, ventricular fibrillation; ↑, increased; ↓, reduced; ↔, no change.

**Table 3:** Effects of kaempferol on myocardial calcium regulation and cardiac rhythm in cardiac injury.

Animals	Model	Dose and Duration of Kaempferol	Findings	Reference
Rats	STZ-induced diabetes	100 mg/kg bw (p.o.) for 45 days	↑ total ATPase, ↑ Na^+^/K^+^-ATPase ↑ Ca^2+^-ATPase	[26]
Mouse cardiomyocytes	LADCA-ligation-induced HF	10 mM for 80 s	↑ mt Ca^2+^ uptake, ↓ diastolic Ca^2+^ waves, ↓ diastolic Ca^2+^ sparks ↓ caffeine-induced Ca^2+^ transients ↔ amplitude of Ca^2+^ transients ↑ tau decay of Ca^2+^ transients ↓ spontaneous AP ↓ resting potentials ↓ ventricular arrythmia	[44]
Primary rat ventricular myocytes	FCCP-induced Ca^2+^ waves	10 μM (post-treatment)	↓ Ca^2+^ waves ↓ basal Ca^2+^	[41]
Mice	High-fat sucrose (obese with T2DM and overweight with insulin resistance)	1 mL/h (i.v.) for 7 days	↓ AF, ↓ P wave duration ↓ SNRT120, ↑ MCUC opening	[50]
C57BL/6 mice	Ang-II-induced cardiac sinus dysfunction	0.5 mmol per kg (s.c) for 3 weeks	↓ sinus pauses	[51]
Langendorff isolated rat heart	Thoracic aortic banding-induced cardiac hypertrophy + ISO-induced arrhythmia	1 μM	↑ ISO-induced PVC ↔ ISO-induced VF frequency ↓ mt Ca^2+^ transient amplitude	[52]
Mouse primary cardiomyocytes	Thoracic aortic banding-induced cardiac hypertrophy. ISO was added to induce arrythmia	10 μM	↔ amplitude of Ca^2+^ transients ↔ tau decay of mt Ca^2+^ transients ↑ mt Ca^2+^ accumulation pace ↓ cytosolic Ca^2+^, ↑ VF frequency ↓ cytosolic spontaneous Ca^2+^ waves latency	[52]
Cardiomyocytes	Caffeine induced Ca^2+^ uptake	10 μM	↑ mt Ca^2+^ uptake	[53]
HL-1 cardiomyocytes	Caffeine induced Ca^2+^ uptake	10 μM	↑ mt Ca^2+^ uptake	[54]
Cardiomyocytes from RyR2^R4496C/WT^ mice	ISO-induced Ca^2+^ waves	10 μM	↓ spontaneous diastolic Ca^2+^ waves	[54]

AF, atrial fibrillation; Ang II, angiotensin II; AP, action potential; FCCP, protonophore carbonyl cyanide p-(trifluoromethoxy)phenylhydrazone; HF, heart failure; ISO, isoprenaline; LADCA, left anterior descending coronary artery; MCUC, mitochondrial Ca^2+^ uniporter complex; mt, mitochondria; p.o., per oral; s.c., subcutaneous; SNRT120, sinus recovery time after 120 ms pacing; T2DM, type 2 diabetes mellitus; PVC, premature ventricular contractions; VF, ventricular fibrillation; ↑, increased; ↓, reduced; ↔, no change.

**Table 4:** Effects of kaempferol on cardiac oxidative stress and inflammation in cardiac injury.

Animals	Model	Dose and Duration of Kaempferol	Findings	Reference
Rats	Acute myocardial infarction	10 mg/kg (i.g.) for 4 weeks	↓ NLRP3, ↓ NLRP3 protein ↓ NLRP3 gene, ↓ GSDMD ↓ GSDMD protein, ↓ GSDMD gene ↓ IL-1β, ↓ IL-1β protein ↓ NF-κB p65 gene ↓ IL-1β gene, ↓ NF-κB p65 protein	[2]
Mice	Aorta-banding-induced cardiac remodeling	100 mg/kg/d (i.g.) for 6 weeks	↓ p-ERK1/2 protein, ↓ p-JNK1/2 protein, ↓ p-p38 protein ↓ p-ASK1 protein, ↓ 4-HNE staining ↑ SOD, ↑ GSH/GSSG	[3]
H9c2 cardiomyocytes	Phenylephrine-induced cell hypertrophy	25 µM for 2 h (pretreatment)	↓ p-ASK1 protein, ↓ p-p38 protein ↓ p-JNK1/2 protein, ↑ SOD activity ↓ oxidative stress, ↑ GSH/GSSG	[3]
Rat cardiomyocytes	Anoxia/Reperfusion	10, 20 or 40 μM for 24 h (pretreatment)	↓ ROS	[4]
H9c2 cardiomyocytes	H/R-induced injury	5, 10, 20, or 30 μM for 2 h (pretreatment)	↓ ROS, ↓ MDA, ↑ SOD activity ↑ GPx activity, ↓ IL-8, ↓ IL-1β ↓ IL-10, ↓ TNF-α, ↓ NO ↓ iNOS activity	[5]
Cardiac fibroblasts	TGF in Ang-II-stimulated	2.5 and 10 mM (pretreatment)	↓ TNF-α, ↓ TNF-α gene, ↓ IL-6 gene ↑ IκB-α protein, ↓ VCAM-1 gene ↓ p-ERK protein, ↓ p-p38 protein ↓ H_2_O_2_ level, ↓ O_2_^−•^ level ↑ p-AMPK protein, ↑ Nrf-2 protein ↑ HO-1 gene ↑ NQO-1 gene In siRNA transfected cells: ↔ NF-κB p65, ↔ IL-6 In siAMPK transfected cells: ↔ Nrf2 protein	[6]
Rats	Doxorubicin-induced cardiac injury	1, 2, 5, 10, and 20 mg/kg/d on alternates for 3 injections (i.p.) (pretreatment)	↑ SOD activity, ↑ catalase activity ↓ p-ERK1/2, ↔ p-p38, ↔ p-JNK	[7]
Mice	Cisplatin-induced cardiotoxicity	10 mg/kg/d (p.o.) for 2 weeks (pretreatment)	↓ IL-6 gene, ↓ IL-6, ↓ HMGB1 gene ↓ TNF-α gene, ↓ TNF-α ↓ p-NF-κB protein, ↓ MCP-1 gene ↓ CD68	[8]
H9c2 cardiomyocytes	Cisplatin-induced cardiotoxicity	1, 5, and 10 μM for 1 h pretreatment	↓ IL-6 gene, ↓ IL-6, ↓ HMGB1 gene ↓ TNF-α gene, ↓ TNF-α ↓ p-NF-κB protein, ↓ MCP-1 gene ↓ p-STING, ↓ p-TBK-1	[8]
Rats	5-Fluorouracil-induced cardiotoxicity	1 mg/kg/d (i.p.) for 14 days (post-treatment)	↔ COX-2, ↑ VEGF ↓ MDA, ↓ total antioxidant capacity	[9]
Rat cardiomyocytes	Clozapine-induced cardiac injury	10, 20, and 50 μM for 4 h	All concentrations: ↑ cell viability, ↓ ROS, ↓ MDA 50 μM: ↑ GSH, ↓ GSSG	[10]
Diabetic rats	ISO-induced heart failure	10 and 20 mg/kg/d (p.o.) for 42 days (pretreatment)	↑ Nrf2 gene, ↑ HO-1 gene ↑ γGCS gene, ↓ Keap-1 gene ↓ MDA, ↑ SOD activity ↑ catalase activity, ↑ GPx activity ↑ GST activity, ↑ GR activity ↓ NF-κB p65 activity, ↓ TNF-α ↓ IL-6, ↓ IL-1β, ↓ p-IKKβ protein ↓ nNF-κB p65 protein ↓ COX-2 protein, ↓ iNOS protein ↓ p-ERK protein, ↓ p-p38 protein ↓ PI3K protein, ↑ p-Akt protein ↑ p-GSK3β protein	[11]
Mice	STZ-induced diabetes	10 mg/kg (p.o) on alternate days for 8 weeks	↓ TNF-α, ↓ F4/80, ↓ 3-NT ↑ IκB-α protein, ↓ DHE ↑ Nrf-2 protein, ↑ NQO-1 protein	[13]
Rat cardiomyocytes	High-glucose-induced cell injury	2.5 µM for 1 h (pretreatment)	↓ TNF-α gene, ↓ IL-6 gene ↓ NF-κB p65 protein, ↓ MDA, ↓ DHE, ↑ IκB-α protein, ↑ SOD activity, ↑ Nrf-2 protein ↑ HO-1 protein, ↑ NQO-1 protein	[13]
Rats	ISO-induced cardiac injury	10 mg/kg/d for 7 days (pretreatment)	↑ SOD activity, ↑ catalase activity ↓ MDA, ↔ GSH level, ↔ TNF-α gene	[25]
Rats	I/R-induced cardiac injury	15 mmol/L for 15 min (pretreatment)	↑ GSH/GSSG, ↓ TNF-α, ↓ MDA ↑ SOD activity, ↑ p-GSK-3β(ser) protein	[27]
Diabetic rats	I/R-induced cardiac injury	20 mg/kg/d (i.p.) for 28 days (pretreatment)	↓ MDA, ↑ GSH, ↑ SOD activity ↑ catalase activity, ↓ NF-κB protein ↑ p-ERK protein, ↓ p-JNK protein ↓ p-p38 protein	[28]
Rats	I/R-induced myocardial injury	20 mg/kg/d (i.p.) for 15 days (pretreatment)	↓ MDA, ↑ GSH, ↑ SOD activity ↑ catalase activity, ↓NF-κB protein ↓ p-ERK1/2 protein ↓ p-JNK protein, ↓ p-p38 protein	[31]
H9c2 cardiomyocytes	I/R-induced injury	1 and 5 μM for 24 h (pretreatment)	↓ ROS, ↓ NOX activity ↑ GSH, ↑ SIRT3 protein	[33]
Rats	ISO-induced cardiac injury	5, 10, and 20 mg/kg/d (i.p.) for 15 days (pretreatment)	20 mg/kg/d: ↓ MDA, ↑ GSH, ↑ SOD activity ↑ catalase activity	[43]
C57BL/6 mice	Ang-II-induced cardiac sinus dysfunction	0.5 mmol per kg (s.c) for 3 weeks	↓ ox-CaMKII	[51]
Mouse primary cardiomyocytes	Thoracic aortic banding-induced cardiac hypertrophy + ISO-induced arrhythmia	10 μmol/L	↑ mt ROS	[52]
Rats	STZ-induced diabetes	100 mg/kg bw (p.o) for 45 days	↓ TBARS, ↓ LOOH, ↑ GSH ↓ conjugated diene, ↑ vitamin C ↑ α-Tocopherol, ↑ SOD activity ↑ Catalase activity, ↑ GPx activity ↑ GST activity	[61]
Cardiac fibroblasts	LPS + ATP-induced inflammation	12.5 and 25 μg/mL for 2 h (pretreatment)	↓ TNF-α, ↓ IL-1β, ↓ IL-6 ↓ IL-18, ↓ p-Akt protein ↓ p-NF-κB p65 protein	[62]
Mice	Hemorrhagic shock	10 mg/kg (i.p.) (pre- and post-treatment)	Pretreatment: ↓ MPO activity, ↓ MDA ↑ SOD activity, ↑ HO-1 protein	[63]

COX-2, cyclooxygenase 2; DHE, dihydroethidium; F4/80, macrophage marker F4/80; γGCS, γ-glutamylcysteine synthetase; GSDMD, gasdermin D; GPx, glutathione peroxidase; GR, glutathione reductase; GSH, reduced glutathione; GSSG, oxidized glutathione; GST, glutathione S-transferase; IκB-α, inhibitor of κB kinase α; HMGB1, high mobility group box 1; HO-1, heme oxygenase 1; H_2_O_2_, hydrogen peroxide; 4-HNE, 4-hydroxynonenal; IL, interleukin; iNOS, inducible nitric oxide synthase; i.p., intraperitoneal; I/R, ischemia/reperfusion; ISO, isoprenaline; Keap-1, Kelch-like ECH-associated protein 1; LOOH, lipid hydroperoxide; LPS, lipopolysaccharide; MCP-1, monocyte chemotactic protein 1; MDA, malondialdehyde; MPO, myeloperoxidase; mt, mitochondria; 3-NT, 3-nitrotyrosine; n, nuclear; NQO-1, NAD(P)H:quinone oxidoreductase 1; p-NF-κB, phosphorylated nuclear factor κB; NO, nitric oxide; NOX, NADPH oxidase; NLRP3, NLR family pyrin domain containing 3; Nrf2, nuclear factor erythroid 2 p45-related factor 2; O_2_^−•^, anion superoxide; ox-CAMKII, oxidized Ca^2+^/calmodulin-dependent protein kinase II; p-Akt, phosphorylated protein kinase B; p-AMPK, phosphorylated 5’ AMP-activated protein kinase; p-ASK1, phosphorylated apoptosis signal-regulating kinase 1; p-ERK, phosphorylated extracellular signal-regulated kinase; p-GSK-3β(ser), phosphorylated glycogen synthase kinase-3β (serine); p-IKKβ, p-IκB kinase beta; p-p38, phosphorylated p38 mitogen-activated protein kinase; p-STING, phosphorylated stimulator of interferon genes; p-TBK, phosphorylated TANK-binding kinase 1; PI3K, phosphoinositide 3-kinase; ROS, reactive oxygen species; siRNA, small interfering RNA; SIRT3, silent information regulator type 3; SOD, superoxide dismutase; STZ, streptozotocin; TBARS, thiobarbituric reactive substance; TGF, transforming growth factor; TNF-α, tumor necrosis factor α; VCAM-1, vascular cell adhesion molecule 1; VEGF, vascular endothelial growth factor; ↑, increased; ↓, reduced; ↔, no change.

**Table 5:** Effects of kaempferol on myocardial mitochondrial function and other organelle damage.

Animals	Model	Dose and Duration of Kaempferol	Findings	Reference
Rat cardiomyocytes	Anoxia/reperfusion	10, 20 or 40 μM for 24 h (pretreatment)	↓ mMP loss, ↓ mPTP opening ↓ cytochrome c release ↑ SIRT1 protein	[4]
H9c2 cardiomyocytes	Doxorubicin-induced injury	20 μM for 4 h (pretreatment)	↓ cytochrome c release ↓ mMP loss	[7]
Rat cardiomyocytes	Clozapine-induced cardiac injury	10, 20, and 50 μM for 4 h	↓ mMP collapse ↓ lysosomal damage	[10]
Rats	I/R-induced cardiac injury	15 mmol/L for 15 min (pretreatment)	↓ cytochrome c release	[27]
H9c2 cardiomyocytes	I/R-induced injury	10 μM for 30 min (pretreatment)	↓ GRP78 protein, ↓ ATF-6α protein ↓ IRE-1α protein, ↓ p-eIF2-α protein ↓ XBP-1 protein, ↓ CHOP protein	[32]
Langendorff isolated heart	I/R-induced injury	10 μM for 20 min pre-ischemia and 50 min post-ischemia	↓ GRP78 protein, ↓ CHOP protein	[32]
Mouse cardiomyocytes	LADCA-ligation-induced HF	10 mM for 80 s	↔ mMP	[44]
Mouse primary cardiomyocytes	Thoracic aortic banding-induced cardiac hypertrophy. ISO was added to induce arrythmia	10 μmol/L	↓ mPTP opening	[52]

ATF6α, activating transcription factor 6α; C/EBP homologous protein (CHOP), eIF2α, eukaryotic initiation factor 2α; GRP78, glucose regulatory protein 78; I/R, ischemia/reperfusion; IRE1α, inositol-requiring transmembrane kinase endoribonuclease-1α; ISO, isoprenaline; LADCA, left anterior descending coronary artery; mito, mitochondria; mMP, mitochondrial membrane potential; mPTP, mitochondria permeability transition pore; SIRT1, silent information regulator X-box binding protein 1 (XBP-1), ↑, increased; ↓, reduced; ↔, no change.

**Table 6:** Effects of kaempferol on myocardial apoptosis in cardiac injury.

Animals	Model	Dose and Duration of Kaempferol	Findings	Reference
Rats	Acute myocardial infarction	10 mg/kg (i.g.) for 4 weeks	↓ TUNEL, ↓ caspase 1, ↓ caspase 1 protein ↓ caspase 1 gene, ↓ ASC protein	[2]
Rat cardiomyocytes	Anoxia/Reperfusion	10, 20 or 40 μM for 24 h (pretreatment)	↓ caspase 3 activity ↓ caspase 3 protein ↓ apoptotic cells, ↑ Bcl-2 protein	[4]
H9c2 cardiomyocytes	H/R-induced injury	5, 10, 20, or 30 μM for 2 h (pretreatment)	↓ apoptosis, ↑ Bcl-2 protein ↓ Bax protein, ↓ miR-21 gene ↓ caspase 3 activity	[5]
Rats	Doxorubicin-induced cardiac injury	10 mg/kg/d on alternates for 3 injections (i.p.) (pretreatment)	↓ TUNEL, ↓ caspase 3 protein ↑ Bcl-2 protein, ↓ Bax protein ↓ Bax gene, ↑ Bcl-2 gene	[7]
H9c2 cardiomyocytes	Doxorubicin-induced injury	20 μM for 4 h (pretreatment)	↓ TUNEL, ↓ PAPR cleavage ↓ caspase 3 protein, ↓ p53 gene ↓ p53 protein, ↔ Fas gene ↑ Bcl-2 protein, ↓ Bax protein ↓ Bax gene, ↑ Bcl-2 gene	[7]
Mice	Cisplatin-induced cardiotoxicity	10 mg/kg/d (p.o.) for 2 weeks (pretreatment)	↓ TUNEL, ↓ Bax protein ↑ Bcl-2 protein	[8]
H9c2 cardiomyocytes	Cisplatin-induced cardiotoxicity	1, 5, and 10 μM for 1 h pretreatment	↓ TUNEL, ↓ Bax protein ↑ Bcl-2 protein	[8]
Diabetic rats	ISO-induced heart failure	10 and 20 mg/kg/d (p.o.) for 42 days (pretreatment)	↓ Bax protein, ↑ Bcl-2 protein ↓ caspase 3, ↓ TUNEL	[11]
Primary and H9c2 rat cardiomyocytes	High-glucose-induced cell injury	2.5 µM for 1 h (pretreatment)	↑ Bcl-2 protein, ↓ Bax protein	[13]
Mice	STZ-induced diabetes	10 mg/kg (p.o) on alternate days for 8 weeks	↑ Bcl-2 protein, ↓ Bax protein ↓ TUNEL	[13]
Rats	I/R-induced cardiac injury	15 mmol/L for 15 min (pretreatment)	↓ apoptotic index ↓ cleaved caspase-3 protein	[27]
Diabetic rats	I/R-induced cardiac injury	20 mg/kg/d (i.p.) for 28 days (pretreatment)	↓ Bax protein, ↑ Bcl-2 protein ↓ caspase 3	[28]
Rats	I/R-induced myocardial injury	20 mg/kg/d (i.p.) for 15 days (pretreatment)	↓ Bax protein, ↑ Bcl-2 protein ↓ caspase 3	[31]
H9c2 cardiomyocytes	I/R-induced injury	10 μM for 30 min (pretreatment)	↑ Bcl-2 protein, ↓ Bax protein ↓ caspase 3 activity, ↓ apoptosis	[32]
Langendorff isolated heart	I/R injury	10 μM for 20 min pre-ischemia and 50 min post-ischemia	↑ Bcl-2 protein, ↓ Bax protein	[32]
H9c2 cardiomyocytes	I/R-induced injury	1 and 5 μM for 24 h (pretreatment)	↓ Bax protein, ↑ Bcl-2 protein	[33]
Mice	LADCA-ligation-induced HF	12 mg/kg/d for 3 days	↔ TUNEL	[44]
Rats	ISO-induced cardiac injury	5, 10, and 20 mg/kg/d (i.p.) for 15 days (pretreatment)	20 mg/kg/d: ↓ Bax protein, ↑ Bcl-2 protein ↑ Bcl-2/Bax protein	[43]
C57BL/6 mice	Ang-II-induced cardiac sinus dysfunction	0.5 mmol per kg (s.c.) for 3 weeks	↓ TUNEL ↓ caspase-3 activity	[51]

Ang II, angiotensin II; ASC, apoptosis-associated speck-like protein containing a CARD; Bcl-2, B-cell lymphoma 2; Bax, Bcl-2-associated X protein; Fas, First apoptosis signal; i.p., intraperitoneal; I/R, ischemia/reperfusion; ISO, isoprenaline; miR-21, microRNA-21; p53, p53 tumor suppressor gene; PAPR, poly(ADP-ribose)polymerase; p.o., per oral; s.c., subcutaneous; TUNEL, and terminal deoxynucleotidyl transferase dUTP nick-end labeling; ↑, increased; ↓, reduced; ↔, no change.

**Table 7:** Effects of kaempferol on myocardial fibrosis in cardiac injury.

Animals	Model	Dose and Duration of Kaempferol	Findings	Reference
Rats	Acute myocardial infarction	10 mg/kg (i.g.) for 4 weeks	↓ collagen volume fraction ↓ fibrosis	[2]
Mice	Aorta-banding-induced cardiac remodeling	100 mg/kg/d (i.g.) for 6 weeks	↓ LV collagen, ↓ fibronectin gene ↓ Col 1 gene, ↓ Col 3 gene ↓ CTGF gene, ↓ TGF-β protein ↓ p-Smad1/5, ↓ p-Smad3	[3]
Cardiac fibroblasts	Ang-II-stimulated	2.5 and 10 mM (pretreatment)	↓ Col 1 and Col 3 protein, ↓ Col 3 gene ↓ TGF-β protein, ↓ TGF-β gene ↓ fibrosis content	[6]
Primary and H9c2 rat cardiomyocytes	High-glucose-induced cell injury	2.5 µM for 1 h (pretreatment)	↓ collagen-4 protein ↓ TGF-β protein	[13]
Mice	STZ-induced diabetes	10 mg/kg (p.o) on alternate days for 8 weeks	↓ collagen-4 protein, ↓ TGF-β protein ↓ Masson-stained connective tissue	[13]
Rats	ISO-induced cardiac injury	10 mg/kg/d for 7 days (pretreatment)	↓ MMP-9	[25]
Mice	Ang-II-induced cardiac dysfunction	10 mg/kg (i.p.) for 2 weeks	↓ LV collagen volume, ↓ Col 1 gene ↓ Col 3 gene, ↓ CTGF gene ↓ TGF-β1 gene, ↓ α-SMA protein ↓ CD31 protein	[30]
Cardiac fibroblasts	TGF-β1-induced fibrosis	50 μM (concurrent)	↓ Col 1 gene, ↓ Col 3 gene ↓ α-SMA, ↑ MMP-1	[30]

Ang II, angiotensin II; CD31, cluster of differentiation 31; Col, collagen; CTGF, connective tissue growth factor; i.g., intragastric; i.p., intraperitoneal; ISO, isoprenaline; LV, left ventricle; MMP, matrix metalloproteinase; p-Smad, phosphorylated small mother against decapentaplegic; α-SMA, α-smooth muscle actin; STZ, streptozotocin; TGF-β, transforming growth factor β; ↑, increased; ↓, reduced.

## Data Availability

Not applicable.

## References

[1] WHO Cardiovascular Diseases. https://www.who.int/health-topics/cardiovascular-diseases#tab=tab_1.2023.

[2] Hua F., Li J.Y., Zhang M., Zhou P., Wang L., Ling T.J., Bao G.H. (2022). Kaempferol-3-O-rutinoside exerts cardioprotective effects through NF-κB/NLRP3/Caspase-1 pathway in ventricular remodeling after acute myocardial infarction. J. Food Biochem..

[3] Feng H., Cao J., Zhang G., Wang Y. (2017). Kaempferol attenuates cardiac hypertrophy via regulation of ASK1/MAPK signaling pathway and oxidative stress. Planta Med..

[4] Guo Z., Liao Z., Huang L., Liu D., Yin D., He M. (2015). Kaempferol protects cardiomyocytes against anoxia/reoxygenation injury via mitochondrial pathway mediated by SIRT1. Eur. J. Pharmacol..

[5] Huang J., Qi Z. (2020). MiR-21 mediates the protection of kaempferol against hypoxia/reoxygenation-induced cardiomyocyte injury via promoting Notch1/PTEN/AKT signaling pathway. PLoS ONE.

[6] Du Y., Han J., Zhang H., Xu J., Jiang L., Ge W. (2019). Kaempferol prevents against Ang II-induced cardiac remodeling through attenuating Ang II-induced inflammation and oxidative stress. J. Cardiovasc. Pharmacol..

[7] Xiao J., Sun G.B., Sun B., Wu Y., He L., Wang X., Chen R.C., Cao L., Ren X.Y., Sun X.B. (2012). Kaempferol protects against doxorubicin-induced cardiotoxicity in vivo and in vitro. Toxicology.

[8] Qi Y., Ying Y., Zou J., Fang Q., Yuan X., Cao Y., Cai Y., Fu S. (2020). Kaempferol attenuated cisplatin-induced cardiac injury via inhibiting STING/NF-κB-mediated inflammation. Am. J. Transl. Res..

[9] Safarpour S., Pirzadeh M., Ebrahimpour A., Shirafkan F., Madani F., Hosseini M., Moghadamnia A.A., Kazemi S. (2022). Protective effect of kaempferol and its nanoparticles on 5-fluorouracil-induced cardiotoxicity in rats. Biomed. Res. Int..

[10] Bakhshii S., Khezri S., Ahangari R., Jahedsani A., Salimi A. (2021). Protection of clozapine-induced oxidative stress and mitochondrial dysfunction by kaempferol in rat cardiomyocytes. Drug Dev. Res..

[11] Zhang L., Guo Z., Wang Y., Geng J., Han S. (2019). The protective effect of kaempferol on heart via the regulation of Nrf2, NF-κβ, and PI3K/Akt/GSK-3β signaling pathways in isoproterenol-induced heart failure in diabetic rats. Drug Dev. Res..

[12] Anamalley R., Rajassageran L., Apparoo Y., Jauri M.H., Kamisah Y., Yunos N.M., Zainalabidin S. (2022). Repeated administration of low dose isoprenaline on the rat’s cardiovascular system. Sains Malays..

[13] Chen X., Qian J., Wang L., Li J., Zhao Y., Han J., Khan Z., Chen X., Wang J., Liang G. (2018). Kaempferol attenuates hyperglycemia-induced cardiac injuries by inhibiting inflammatory responses and oxidative stress. Endocrine.

[14] Leuci R., Brunetti L., Poliseno V., Laghezza A., Loiodice F., Tortorella P., Piemontese L. (2020). Natural compounds for the prevention and treatment of cardiovascular and neurodegenerative diseases. Foods.

[15] Dias M.C., Pinto D.C.G.A., Silva A.M.S. (2021). Plant flavonoids: Chemical characteristics and biological activity. Molecules.

[16] Shen N., Wang T., Gan Q., Liu S., Wang L., Jin B. (2022). Plant flavonoids: Classification, distribution, biosynthesis, and antioxidant activity. Food Chem..

[17] Wei Q., Li Q.Z., Wang R.L. (2023). Flavonoid components, distribution, and biological activities in *Taxus*: A review. Molecules.

[18] Siti H.N., Jalil J., Asmadi A.Y., Kamisah Y. (2021). Rutin modulates MAPK pathway differently from quercetin in angiotensin II-induced H9c2 cardiomyocyte hypertrophy. Int. J. Mol. Sci..

[19] Li L., Luo W., Qian Y., Zhu W., Qian J., Li J., Jin Y., Xu X., Liang G. (2019). Luteolin protects against diabetic cardiomyopathy by inhibiting NF-κB-mediated inflammation and activating the Nrf2-mediated antioxidant responses. Phytomedicine.

[20] Meng C., Guo Z., Li D., Li H., He J., Wen D., Luo B. (2018). Preventive effect of hesperidin modulates inflammatory responses and antioxidant status following acute myocardial infarction through the expression of PPAR-γ and Bcl-2 in model mice. Mol. Med. Rep..

[21] Newman D.J., Cragg G.M. (2020). Natural products as sources of new drugs over the nearly four decades from 01/1981 to 09/2019. J. Nat. Prod..

[22] Calderón-Montaño J.M., Burgos-Morón E., Pérez-Guerrero C., López-Lázaro M. (2011). A review on the dietary flavonoid kaempferol. Mini Rev. Med. Chem..

[23] Mustafa N.H., Jalil J., Saleh M.S.M., Zainalabidin S., Asmadi A.Y., Kamisah Y. (2023). *Parkia speciosa* Hassk. empty pod extract prevents cardiomyocyte hypertrophy by inhibiting MAPK and calcineurin-NFATC3 signaling pathways. Life.

[24] Wang X., Yang Y., An Y., Fang G. (2019). The mechanism of anticancer action and potential clinical use of kaempferol in the treatment of breast cancer. Biomed. Pharmacother..

[25] Vishwakarma A., Singh T.U., Rungsung S., Kumar T., Kandasamy A., Parida S., Lingaraju M.C., Kumar A., Kumar A., Kumar D. (2018). Effect of kaempferol pretreatment on myocardial injury in rats. Cardiovasc. Toxicol..

[26] Al-Numair K.S., Veeramani C., Alsaif M.A., Chandramohan G. (2015). Influence of kaempferol, a flavonoid compound, on membrane-bound ATPases in streptozotocin-induced diabetic rats. Pharm. Biol..

[27] Zhou M., Ren H., Han J., Wang W., Zheng Q., Wang D. (2015). Protective effects of kaempferol against myocardial ischemia/reperfusion injury in isolated rat heart via antioxidant activity and inhibition of glycogen synthase kinase-3β. Oxid. Med. Cell. Longev..

[28] Suchal K., Malik S., Khan S.I., Malhotra R.K., Goyal S.N., Bhatia J., Ojha S., Arya D.S. (2017). Molecular pathways involved in the amelioration of myocardial injury in diabetic rats by kaempferol. Int. J. Mol. Sci..

[29] Zhou W., Peng C., Wang D., Li J., Tu Z., Zhang L. (2022). Interaction mechanism between OVA and flavonoids with different hydroxyl groups on B-ring and effect on antioxidant activity. Foods.

[30] Liu Y., Gao L., Guo S., Liu Y., Zhao X., Li R., Yan X., Li Y., Wang S., Niu X. (2017). Kaempferol alleviates angiotensin II-induced cardiac dysfunction and interstitial fibrosis in mice. Cell. Physiol. Biochem..

[31] Suchal K., Malik S., Gamad N., Malhotra R.K., Goyal S.N., Chaudhary U., Bhatia J., Ojha S., Arya D.S. (2016). Kaempferol attenuates myocardial ischemic injury via inhibition of MAPK signaling pathway in experimental model of myocardial ischemia-reperfusion injury. Oxid. Med. Cell. Longev..

[32] Kim D.S., Ha K.C., Kwon D.Y., Kim M.S., Kim H.R., Chae S.W., Chae H.J. (2008). Kaempferol protects ischemia/reperfusion-induced cardiac damage through the regulation of endoplasmic reticulum stress. Immunopharmacol. Immunotoxicol..

[33] Sun C., Wang T., Wang C., Zhu Z., Wang X., Xu J., An H. (2022). The protective effect of kaempferol against ischemia/reperfusion injury through activating SIRT3 to inhibit oxidative stress. Braz. J. Cardiovasc. Surg..

[34] Goetze J.P., Bruneau B.G., Ramos H.R., Ogawa T., de Bold M.K., de Bold A.J. (2020). Cardiac natriuretic peptides. Nat. Rev. Cardiol..

[35] Frantz S., Hundertmark M.J., Schulz-Menger J., Bengel F.M., Bauersachs J. (2022). Left ventricular remodelling post-myocardial infarction: Pathophysiology, imaging, and novel therapies. Eur. Heart J..

[36] Dang A., Zheng D., Wang B., Zhang Y., Zhang P., Xu M., Liu G., Liu L. (1999). The role of the renin-angiotensin and cardiac sympathetic nervous systems in the development of hypertension and left ventricular hypertrophy in spontaneously hypertensive rats. Hypertens. Res..

[37] Pedreanez A., Mosquera J., Munoz N., Robalino J., Tene D. (2022). Diabetes, heart damage, and angiotensin II. What is the relationship link between them? A minireview. Endocr. Regul..

[38] Al-Numair K.S., Chandramohan G., Veeramani C., Alsaif M.A. (2015). Ameliorative effect of kaempferol, a flavonoid, on oxidative stress in streptozotocin-induced diabetic rats. Redox Rep..

[39] Rendra E., Riabov V., Mossel D.M., Sevastyanova T., Harmsen M.C., Kzhyshkowska J. (2019). Reactive oxygen species (ROS) in macrophage activation and function in diabetes. Immunobiology.

[40] Hsiao F.C., Wang C.L., Chang P.C., Lu Y.Y., Huang C.Y., Chu P.H. (2020). Angiotensin receptor neprilysin inhibitor for patients with heart failure and reduced ejection fraction: Real-world experience from Taiwan. J. Cardiovasc. Pharmacol. Ther..

[41] Zhao Z., Gordan R., Wen H., Fefelova N., Zang W.J., Xie L.H. (2013). Modulation of intracellular calcium waves and triggered activities by mitochondrial ca flux in mouse cardiomyocytes. PLoS ONE.

[42] Hudson S., Pettit S. (2020). What is ‘normal’ left ventricular ejection fraction?. Heart.

[43] Suchal K., Malik S., Gamad N., Malhotra R.K., Goyal S.N., Bhatia J., Arya D.S. (2016). Kampeferol protects against oxidative stress and apoptotic damage in experimental model of isoproterenol-induced cardiac toxicity in rats. Phytomedicine.

[44] Hagiwara H., Watanabe M., Fujioka Y., Kadosaka T., Koizumi T., Koya T., Nakao M., Kamada R., Temma T., Okada K. (2022). Stimulation of the mitochondrial calcium uniporter mitigates chronic heart failure-associated ventricular arrhythmia in mice. Heart Rhythm.

[45] Reddy Y.N.V., El-Sabbagh A., Nishimura R.A. (2018). Comparing pulmonary arterial wedge pressure and left ventricular end diastolic pressure for assessment of left-sided filling pressures. JAMA Cardiol..

[46] Kamisah Y., Hassan H.H.C. (2023). Therapeutic use and molecular aspects of ivabradine in cardiac remodeling: A review. Int. J. Mol. Sci..

[47] Kakehi K., Iwanaga Y., Watanabe H., Sonobe T., Akiyama T., Shimizu S., Yamamoto H., Miyazaki S. (2019). Modulation of sympathetic activity and innervation with chronic ivabradine and β-blocker therapies: Analysis of hypertensive rats with heart failure. J. Cardiovasc. Pharmacol. Ther..

[48] Hamilton S., Terentyev D. (2022). ER stress and calcium-dependent arrhythmias. Front. Physiol..

[49] Salim S., Yunos N., Jauri M., Kamisah Y. (2020). Cardiotonic effects of cardiac glycosides from plants of Apocynaceae family. Chula. Med. J..

[50] Fossier L., Panel M., Butruille L., Colombani S., Azria L., Woitrain E., Decoin R., Torrente A.G., Thireau J., Lacampagne A. (2022). Enhanced mitochondrial calcium uptake suppresses atrial fibrillation associated with metabolic syndrome. J. Am. Coll. Cardiol..

[51] An M., Kim M. (2015). Protective effects of kaempferol against cardiac sinus node dysfunction via CaMKII deoxidization. Anat. Cell. Biol..

[52] Hamilton S., Terentyeva R., Kim T.Y., Bronk P., Clements R.T., O-Uchi J., Csordás G., Choi B.R., Terentyev D. (2018). Pharmacological modulation of mitochondrial Ca^2+^ content regulates sarcoplasmic reticulum Ca^2+^ release via oxidation of the ryanodine receptor by mitochondria-derived reactive oxygen species. Front. Physiol..

[53] Sander P., Feng M., Schweitzer M.K., Wilting F., Gutenthaler S.M., Arduino D.M., Fischbach S., Dreizehnter L., Moretti A., Gudermann T. (2021). Approved drugs ezetimibe and disulfiram enhance mitochondrial Ca2+ uptake and suppress cardiac arrhythmogenesis. Br. J. Pharmacol..

[54] Schweitzer M.K., Wilting F., Sedej S., Dreizehnter L., Dupper N.J., Tian Q., Moretti A., My I., Kwon O., Priori S.G. (2017). Suppression of arrhythmia by enhancing mitochondrial Ca^2+^ uptake in catecholaminergic ventricular tachycardia models. JACC Basic Transl. Sci..

[55] Lombardi A.A., Gibb A.A., Arif E., Kolmetzky D.W., Tomar D., Luongo T.S., Jadiya P., Murray E.K., Lorkiewicz P.K., Hajnóczky G. (2019). Mitochondrial calcium exchange links metabolism with the epigenome to control cellular differentiation. Nat. Commun..

[56] Lai L., Qiu H. (2020). The physiological and pathological roles of mitochondrial calcium uptake in heart. Int. J. Mol. Sci..

[57] Eisner D.A., Caldwell J.L., Trafford A.W., Hutchings D.C. (2020). The control of diastolic calcium in the heart: Basic mechanisms and functional implications. Circ. Res..

[58] Siti H.N., Kamisah Y., Kamsiah J. (2015). The role of oxidative stress, antioxidants and vascular inflammation in cardiovascular disease (a review). Vascul. Pharmacol..

[59] Gui J.S., Mustafa N.H., Jalil J., Jubri Z., Kamisah Y. (2019). Modulation of NOX4 and MAPK signalling pathways by *Parkia speciosa* empty pods in H9c2 cardiomyocytes exposed to H_2_O_2_. Indian J. Pharm. Sci..

[60] Gui J.S., Jalil J., Jubri Z., Kamisah Y. (2019). *Parkia speciosa* empty pod extract exerts anti-inflammatory properties by modulating NFκB and MAPK pathways in cardiomyocytes exposed to tumor necrosis factor-α. Cytotechnology.

[61] Mustafa N.H., Ugusman A., Jalil J., Kamisah Y. (2018). Anti-inflammatory property of *Parkia speciosa* empty pod extract in human umbilical vein endothelial cells. J. Appl. Pharm. Sci..

[62] Tang X.L., Liu J.X., Dong W., Li P., Li L., Hou J.C., Zheng Y.Q., Lin C.R., Ren J.G. (2015). Protective effect of kaempferol on LPS plus ATP-induced inflammatory response in cardiac fibroblasts. Inflammation.

[63] Yang Q.S., He L.P., Zhou X.L., Zhao Y., Shen J., Xu P., Ni S.Z. (2015). Kaempferol pretreatment modulates systemic inflammation and oxidative stress following hemorrhagic shock in mice. Chin. Med..

[64] Tonelli C., Chio I.I.C., Tuveson D.A. (2018). Transcriptional regulation by Nrf2. Antioxid. Redox Signal..

[65] Syamsunarno M.R.A., Safitri R., Kamisah Y. (2021). Protective effects of *Caesalpinia sappan* Linn. and its bioactive compounds on cardiovascular organs. Front. Pharmacol..

[66] Almowallad S., Alqahtani L.S., Mobashir M. (2022). NF-kB in signaling patterns and its temporal dynamics encode/decode human diseases. Life.

[67] Beckendorf J., van den Hoogenhof M.M.G., Backs J. (2018). Physiological and unappreciated roles of CaMKII in the heart. Basic Res. Cardiol..

[68] Peoples J.N., Saraf A., Ghazal N., Pham T.T., Kwong J.Q. (2019). Mitochondrial dysfunction and oxidative stress in heart disease. Exp. Mol. Med..

[69] Zorova L.D., Popkov V.A., Plotnikov E.Y., Silachev D.N., Pevzner I.B., Jankauskas S.S., Babenko V.A., Zorov S.D., Balakireva A.V., Juhaszova M. (2018). Mitochondrial membrane potential. Anal. Biochem..

[70] Sabbah H.N., Zhang K., Gupta R.C., Xu J., Singh-Gupta V. (2020). Effects of angiotensin-neprilysin inhibition in canines with experimentally induced cardiorenal syndrome. J. Card. Fail..

[71] Tang B.L. (2016). Sirt1 and the mitochondria. Mol. Cells.

[72] Ren J., Bi Y., Sowers J.R., Hetz C., Zhang Y. (2021). Endoplasmic reticulum stress and unfolded protein response in cardiovascular diseases. Nat. Rev. Cardiol..

[73] Meyer B.A., Doroudgar S. (2020). ER stress-induced secretion of proteins and their extracellular functions in the heart. Cells.

[74] Yang C., Wang X. (2021). Lysosome biogenesis: Regulation and functions. J. Cell. Biol..

[75] Ghazal N., Peoples J.N., Mohiuddin T.A., Kwong J.Q. (2021). Mitochondrial functional resilience after TFAM ablation in the adult heart. Am. J. Physiol. Cell. Physiol..

[76] Ong S.B., Hausenloy D.J. (2017). Mitochondrial dynamics as a therapeutic target for treating cardiac diseases. Handb. Exp. Pharmacol..

[77] Bock F.J., Tait S.W.G. (2020). Mitochondria as multifaceted regulators of cell death. Nat. Rev. Mol. Cell. Biol..

[78] Xu X., Lai Y., Hua Z.C. (2019). Apoptosis and apoptotic body: Disease message and therapeutic target potentials. Biosci. Rep..

[79] Santos-Gallego C.G., Vahl T.P., Goliasch G., Picatoste B., Arias T., Ishikawa K., Njerve I.U., Sanz J., Narula J., Sengupta P.P. (2016). Sphingosine-1-phosphate receptor agonist fingolimod increases myocardial salvage and decreases adverse postinfarction left ventricular remodeling in a porcine model of ischemia/reperfusion. Circulation.

[80] Alex L., Russo I., Holoborodko V., Frangogiannis N.G. (2018). Characterization of a mouse model of obesity-related fibrotic cardiomyopathy that recapitulates features of human heart failure with preserved ejection fraction. Am. J. Physiol. Heart Circ. Physiol..

[81] Ramli F.F., Hashim S.A.S., Raman B., Mahmod M., Kamisah Y. (2022). Role of trientine in hypertrophic cardiomyopathy: A review of mechanistic aspects. Pharmaceuticals.

[82] Ishii R., Okumura K., Akazawa Y., Malhi M., Ebata R., Sun M., Fujioka T., Kato H., Honjo O., Kabir G. (2020). Heart rate reduction improves right ventricular function and fibrosis in pulmonary hypertension. Am. J. Respir. Cell. Mol. Biol..

[83] Ma Z.G., Yuan Y.P., Wu H.M., Zhang X., Tang Q.Z. (2018). Cardiac fibrosis: New insights into the pathogenesis. Int. J. Biol. Sci..

[84] Frangogiannis N. (2020). Transforming growth factor-β in tissue fibrosis. J. Exp. Med..

[85] Mustafa N.H., Jalil J., Zainalabidin S., Saleh M.S.M., Asmadi A.Y., Kamisah Y. (2022). Molecular mechanisms of sacubitril/valsartan in cardiac remodeling. Front. Pharmacol..

[86] Mia M.M., Cibi D.M., Ghani S.A.B.A., Singh A., Tee N., Sivakumar V., Bogireddi H., Cook S.A., Mao J., Singh M.K. (2022). Loss of Yap/Taz in cardiac fibroblasts attenuates adverse remodelling and improves cardiac function. Cardiovasc. Res..

